# Teprotumumab as a Novel Therapy for Thyroid-Associated Ophthalmopathy

**DOI:** 10.3389/fendo.2020.610337

**Published:** 2020-12-17

**Authors:** Terry J. Smith

**Affiliations:** ^1^Department of Ophthalmology and Visual Sciences, Kellogg Eye Center, Ann Arbor, MI, United States; ^2^Division of Metabolism, Endocrinology, and Diabetes, Department of Internal Medicine, University of Michigan Medical School, Ann Arbor, MI, United States

**Keywords:** autoimmune, insulin-like growth factor I receptor, monoclonal antibody, Graves’ disease, thyroid-associated ophthalmopathy, connective tissue

## Abstract

Thyroid-associated ophthalmopathy (TAO) has remained a vexing and poorly managed autoimmune component of Graves’ disease where the tissues surrounding the eye and in the upper face become inflamed and undergo remodeling. This leads to substantial facial disfigurement while in its most severe forms, TAO can threaten eye sight. In this brief paper, I review some of the background investigation that has led to development of teprotumumab as the first and only US FDA approved medical therapy for TAO. This novel treatment was predicated on recognition that the insulin-like growth factor I receptor plays an important role in the pathogenesis of TAO. It is possible that a similar involvement of that receptor in other autoimmune disease may lead to additional indications for this and alternative insulin-like growth factor I receptor-inhibiting strategies.

## Introduction

Thyroid-associated ophthalmopathy (TAO), also known as thyroid eye disease or Graves’ orbitopathy, remains a vexing disease process most frequently occurring in individuals with Graves’ disease (GD) (1). Less commonly, TAO can occur in patients with Hashimoto’s thyroiditis. Autoimmunity similar to that found in the thyroid is presumed to underlie TAO, although responses to the specific autoantigen(s) in the two tissues may not be identical. The medical treatment of TAO has been woefully inadequate, in large part as a consequence of our poor understanding of its pathogenesis. Thus, until very recently, no drug had been approved by the U.S. Food and Drug Administration (FDA) for TAO. TAO can present with a variety of physical signs and symptoms, many of which are shared with other more common diseases of the tissues surrounding the eye. In this brief review, I attempt to provide the historical background underpinning efforts to identify an effective and safe medical therapy for TAO. That direction of study has yielded substantial evidence for meaningful involvement of the insulin-like growth factor-I receptor (IGF-IR) in the development of TAO (2). At the heart of this evidence is the over-expression of IGF-IR by orbital fibroblasts, T and B cells (3–5), the generation of autoantibodies targeting the receptor in patients with GD (3, 6), and the apparent physical and functional collaboration between IGF-IR and the thyrotropin receptor (TSHR) (7). Based nearly entirely on a series of studies conducted *in vitro*, a *β* arrestin-biased agonist that acts as an IGF-IR inhibitor, teprotumumab, now marketed as Tepezza, was repurposed from its initially intended clinical use as an anti-neoplastic agent, to its consideration as a medical therapy for TAO (8). On the strength of two successful clinical trials involving patients with active, moderate to severe disease, teprotumumab has recently been approved by the FDA for use in TAO (9). This approval has therefore ushered in to clinical practice a new era for medically managing this serious and historically underserved manifestation of thyroid autoimmunity.

## Current Understanding of TAO Pathogenesis

**Figure 1:**
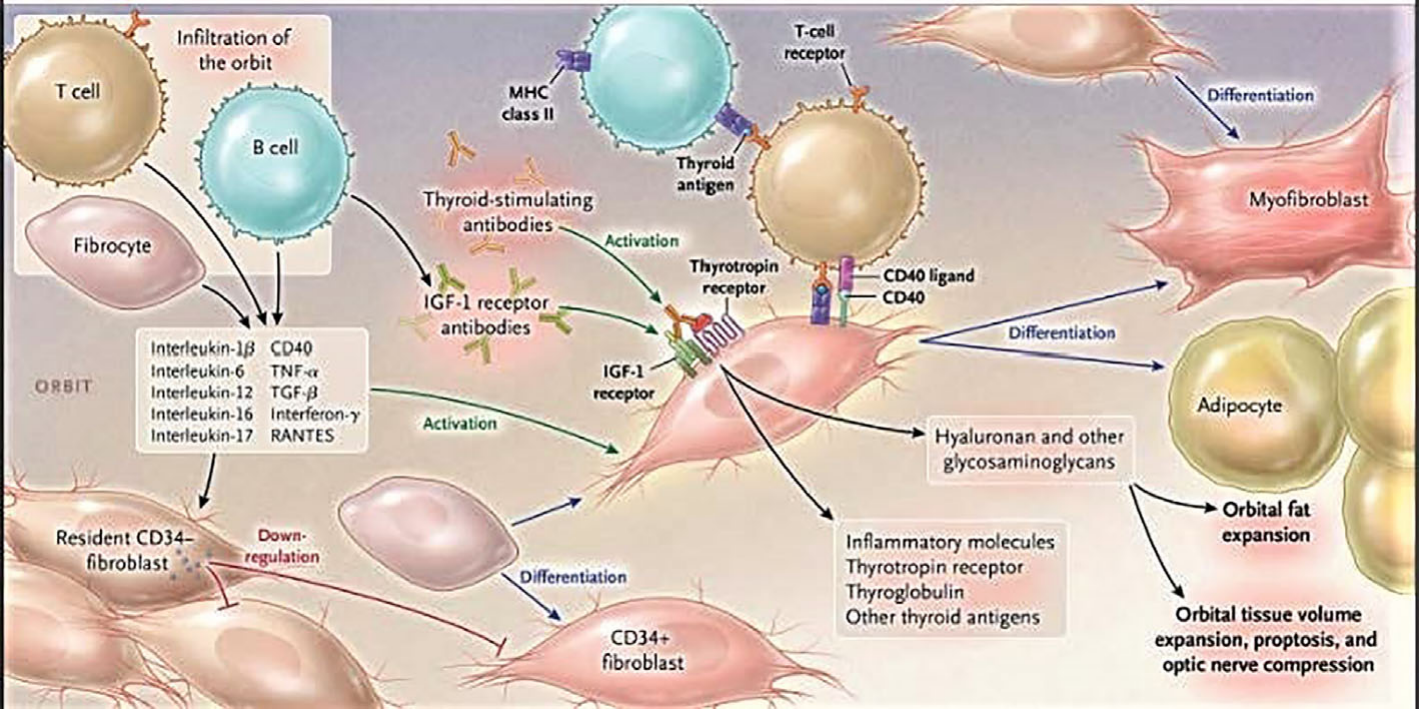
Cartoon proposed model of thyroid-associated ophthalmopathy (TAO) pathogenesis. At the center of the disease are orbital fibroblasts which exhibit particularly robust responses to inflammatory mediators. Among them are CD34^+^ cells which we propose derive from fibrocytes, monocyte-derived progenitor cells trafficked from bone marrow. Fibrocytes circulate in Graves’ disease at higher frequency than that found in healthy individuals. When cultivated from the peripheral circulation, fibrocytes express several thyroid-specific proteins, including thyrotropin receptor (TSHR), thyroglobulin, thyroperoxidase, and sodium-iodide symporter. They also express Class II major histocompatibility complex (MHC) when unstimulated by interferon *γ* and can present antigens. When exposed to the appropriate culture conditions, they undergo differentiation into myofibroblasts (through Smad pathway activation by TGF-β) and adipocytes (through the activation of PPAR-*γ*). Many of the genes expressed by fibrocytes are detected at considerably lower levels in CD34^+^ orbital fibroblasts. We have found that these lower levels of expression result from the actions of Slit2. When activated, CD34^+^ fibrocytes and CD34^+^ fibroblasts generate several pro-inflammatory or anti-inflammatory cytokines, including interleukins 1β, 6, 8, 10, 12, 16, tumor necrosis factor α, and regulated on activation, normal T expressed and secreted (RANTES), CXCL-12 and CD40-CD154. Both CD34^+^ and CD34^-^ orbital fibroblasts cell-surface display insulin-like growth factor-I receptor (IGF-IR). Orbital fibroblasts express three mammalian hyaluronan synthase isoenzymes and UDP glucose dehydrogenase and synthesize hyaluronan, the glycosaminoglycan associated with expanding orbital tissue in TAO. The vast majority of hyaluronan synthesis occurs in CD34^-^ orbital fibroblasts. From N. Engl. J. Med, Smith T.J. and Hegedus L., Graves’ Disease, 375; 1552-1565. Copyright ^©^ (2016) Massachusetts Medical Society. Reprinted with permission.

A number of key insights have been generated in the recent past by several laboratory groups across North America and abroad. At the heart of TAO is the growing and sometimes reluctant recognition that orbital fibroblasts from diseased orbits (GD-OF) comprise a heterogeneous cell population and are involved in pathogenesis as a consequence of the unique presence of CD34^+^ cells where CD34 indicates a cell phenotype with specific characteristics (10–14). These cells have been proposed as the dominant mediators of TAO development by virtue of their extraordinary responsiveness to inflammatory mediators such as cytokines and growth factors (15–19) (**Figure 1**). They express key promoters of inflammation including both prostaglandin endoperoxide H synthase 1 and 2, the latter of which is highly inducible (15, 16, 20, 21) and 15-lipoxygenase (22). We contend that the identification of CD34^+^CXCR4^+^Col I^+^ fibroblasts in the TAO orbit as a discrete subset of GD-OF and their putative derivation from circulating fibrocytes represents a plausible explanation for the markedly heterogeneous behavior found among these disease-derived fibroblasts (14). The aggregate of these markers identifies fibrocytes and distinguishes them from fibroblasts and other cell types (23). These cells can undergo adipogenic differentiation (24). The Thy-1^−^ subset of GD-OF is particularly susceptible to pro-adipogenic factors while those of the Thy-1^+^ phenotype can undergo differentiation into myofibroblasts through the actions of TGF-β and the activation of the Smad pathway (25, 26). CD34^+^ OF exhibit unique phenotypic attributes which can be attributed, at least in part, to their promiscuous expression of the autoimmune regulator protein (27). They synthesize several proteins, the expressions of which were previously thought to be restricted to the thyroid, including TSHR, thyroglobulin, thyroperoxidase, and sodium iodide symporter (28, 29). But the expression of tissue-specific proteins that behave as autoantigens is not limited to those relevant to the thyroid. Fibrocytes have also been found to express those protein antigens implicated in type 1 diabetes mellitus (29). Fibrocytes cross-talk with T cells, and especially with those polarized to the Th17 paradigm, the cytokines from which appear to play important roles in driving orbital inflammation in TAO (30). IL-17A promotes the expression of regulated on activation, normal T cell expressed and secreted (RANTES, CCL5) by orbital fibroblasts, effects mediated through the CD40/CD154 (CD40 ligand) bridge (31).

GD-OF and fibrocytes have been found to express TSHR (14, 32). This receptor was subsequently found to be functional and could mediate effects of both TSH and thyroid-stimulating autoantibodies (7, 14). Activation of several genes, including those encoding cytokines implicated in the pathogenesis of TAO, has been reported (19, 33–35). Fibrocytes are trafficked to sites of tissue injury through several chemokine networks, most notably the CXCL-12/CXCR4 pathway (36) which is under the control of TSHR signaling. A potentially important molecular conduit, the activation of which can influence gene expression in GD-OF, is the CD40–CD154 bridge (21, 37). Ligation of CD40 displayed on the surface of these cells results in substantial upregulation of hyaluronan synthesis and the generation of prostaglandin E_2_ through the induction of prostaglandin endoperoxide H synthase 2.

## TAO has Long Been a Disease the Treatment for Which Exemplifies Inadequacy

Despite its initial published descriptions, dating to the 19^th^ century (38), precious little progress had been made in identifying safe and effective medical therapy for TAO (39, 40). Of the available medications, glucocorticoids have been the most widely used. Standardization of their use, including indications, exclusions, treatment duration, and dosages has yet to be established with placebo-controlled, prospective, and adequately powered clinical trials. Some clinicians taking care of patients with TAO extoll them as the “gold standard”, but the basis for this often strongly expressed opinion appears to rest on dubious grounds rather than on firm scientific evidence. Both oral and intravenous routes of steroid administration have been advocated. More recent reports suggest that IV pulse may be more effective and associated with fewer side effects than those delivered by the oral route (41). But even staunch advocates of steroid use in TAO typically concede that these agents fail to consistently provide benefit to patients and that only 50% respond meaningfully (42). Further, many experts acknowledge that responses to steroids are essentially limited to amelioration of inflammatory signs and symptoms. Among the most informative reports of steroid use in active, moderate to severe TAO, was that of Bartalena and colleagues (43). They examined three different cumulative dosages (2.25, 4.98, and 7.47 g) administered as 12 weekly infusions. Their study demonstrated that short-term response, as measured by improvement in the clinical activity score (CAS), was greatest in those receiving the highest dosage. Patients receiving 7.47 g also showed a 0.6 mm proptosis reduction at 12 weeks compared to baseline, a result widely considered to be lacking clinical importance. Evidence suggests that glucocorticoids in combination with external beam radiation may be somewhat superior to steroids alone (44–47). Other agents have been proposed for combination with steroids, including rituximab, mycophenolate and azathioprine (48–50). In my view, whether used as a single agent or in combination with radiotherapy, systemic steroids should play a progressively less frequent role in the treatment of TAO. In distinction, locally administered steroids, such as those delivered in eye drops or intraorbital injections, may have some utility, especially in mild but symptomatic disease. Steroid therapy in TAO poses substantial risk. Given their limited efficacy, these agents are deemed unsafe since they are associated with several potentially serious side-effects, including hypertension, bone loss, psychiatric illness, and peptic ulcer disease. Moreover, high-dosage intravenous steroids can deleteriously affect liver function and in rare cases, result in fatal hepatic failure (48, 51, 52). Having articulated these objections to steroid use, several biological agents are currently under development or already have been repurposed for potential use in severe TAO. Among them tocilizumab, an IL-6 receptor antagonist, has been subjected to a placebo controlled trial of 32 patients with steroid-resistant TAO at 10 performance sites in Spain (53). Drug or placebo was administered at weeks 0, 4, 8, and 12. Of those receiving active drug, 93.3% met the primary response of a ≥2 point reduction in CAS at week 16 while 58.8% of those treated with placebo met the response (p = 0.04). Notably, a 1.5 mm reduction in proptosis was detected in those receiving the active drug *versus* 0.0 mm in the placebo group at week 16. Observations made later in the study revealed that the small change in proptosis seen with tocilizumab was not durable. A clinical trial of belimumab is currently underway for TAO (54). In addition, preliminary studies examined the potential for immune retolerizing of TSHR in GD and TAO (55).

## IGF-IR and its Signaling Pathway as Therapeutically Exploitable Targets

The IGF-I pathway is complex, comprising several molecules that either enhance signaling initiated by IGF-I or play inhibitory/modulatory roles. IGF-I influences regulation of both normal and abnormal development, metabolism/energy expenditure and immune surveillance (56, 57). It is generated either by the liver or in peripheral tissues. In the latter, it can act locally. IGF binding proteins (IGF-IBP), of which six have been identified, can enhance the half-life of IGF-I within the circulation (58) and can modulate the interactions between IGF-I and IGF-IR (59). The actions of IGF-I and IGF-II are mediated through their interactions with the two cell surface receptors, IGF-IR and IGF-IIR/mannose-6-phosphate receptor (60). IGF-IR is a member of the insulin receptor (IR) family of cell surface-displayed tyrosine kinases. We had suggested some time ago that this pathway might be exploitable for the therapeutic targeting of autoimmune diseases, including GD and TAO (61). The first clue that the IGF-IR pathway might be involved in TAO emanated from the laboratory of Kendall–Taylor nearly three decades ago (62). They reported that IgGs found in sera of patients with TAO could compete for binding of radiolabeled IGF-I to the surface of orbital fibroblasts collected from patients with the disease. While the identity of the binding site was surmised to be IGF-IR by that group, results in a later study conducted by those in another laboratory definitively demonstrated that IGF-IR harbored the binding site and therefore the epitope for autoantibodies circulating in TAO (3). These later studies indicated that IgGs from patients with GD (GD-IgG) could initiate signaling and that the PI3 kinase/FRAP/mTor/p70^S6k^ and Erk p42/44 pathways become activated in GD-OF (3, 6) and thyroid epithelial cells (63). Those studies failed to examine whether GD-IgGs could initiate autophosphorylation of tyrosine residues intrinsic to IGF-IR. Their findings did however raise the possibility that GD-IgG was initiating signaling directly through interactions with IGF-IR. Subsequently, several laboratory groups reexamined this question that GD-IgGs activate IGF-IR autophosphorylation. One group reported that GD-IgGs from a subset of patients could activate receptor kinase activity above levels seen with control (healthy) IgGs (64). In contrast, other investigators have failed to detect GD-IgG-stimulation of IGF-IR kinase activity (65, 66). The concept of anti-IGF-IR antibodies playing a role in TAO has been vigorously argued against by some investigators [summarized in ref (2)]. Their viewpoints have relied heavily on selective reviews of the literature rather than on comprehensive consideration and reconciliation of opposing opinions. The discrepancy between the findings generated by several laboratory groups has yet to be explained satisfactorily. It should be noted that the methods used for detecting IGF-IR activity have varied widely, experimental conditions have not been standardized, and cell treatment protocols have generally relied on single time points, potentially inappropriate target cells, and the uniform absence of assessing endogenous IGF-I concentrations in the culture media. GD-IgG was found to induce the synthesis of hyaluronan in orbital fibroblasts from patients with TAO but not in orbital fibroblasts from healthy individuals (67). Those effects could be mimicked by recombinant IGF-I but not by recombinant human TSH. Subsequent to the detection of anti-IGF-IR autoantibodies in GD and TAO, Tsui et al. reported that IGF-IR and TSHR co-localize in thyroid epithelial cells, orbital fibroblasts and *in situ* in orbital fat (7). Further, in pull-down studies, these investigators found that monoclonal antibodies directed against either receptor protein could precipitate both. They also found that the IGF-IR inhibitory monoclonal antibody, 1H7, could attenuate the activation of Erk 42/44 MAPK initiated by rhTSH, GD-IgG and IGF-I. Those results suggested that signaling emanating from either receptor depended on the activation of IGF-IR. Another report demonstrated that the cellular distribution of IGF-IR was altered in fibroblasts derived from TSHR-null mice (68). A similar pattern of response to disease-derived IgGs was subsequently observed in synovial fibroblasts from patients with rheumatoid arthritis (69). Based on the aggregate of findings implicating IGF-IR in the pathogenesis of TAO, the receptor became a plausible therapeutic target. Further it was suggested that multiple autoimmune diseases, besides GD and rheumatoid arthritis, might share IGF-IR-dependent disease mechanisms and that therapeutic development could be successfully focused on that receptor (61).

## Two Clinical Trials of Teprotumumab Reveal an Effective and Safe Therapy for Moderate to Severe, Active TAO

### Phase 2 Trial

Based on the preclinical studies outlined above, which demonstrated the plausibility of IGF-IR inhibition as a potential treatment strategy for GD and TAO, an initial trial was organized by River Vision Development Company beginning in 2010. The phase 2 study of teprotumumab (RV001, R1507), a fully human monoclonal *β*-arrestin biased agonist IGF-IR inhibitor, involved the repurposing of that drug from its initially intended use in cancer where it had proven ineffective in treating several disease types (70, 71). This trial was a double-masked, randomized, placebo-controlled and multi-centered study with 15 performance sites in North America and Europe (72). Enrolment commenced on 2 July, 2013 and was completed 23 September, 2015. Exclusionary factors included uncontrolled hyper- and hypothyroidism, a history of receiving >1 g of steroids for the treatment of TAO, prior rehabilitative ocular surgeries for TAO and prior treatment with either rituximab or tocilizumab. A total of 88 patients, age range from 18 to 75 years, with moderate to severe, active [clinical activity scores (CAS) ≥4 on a 7-point scale] TAO were randomized to receive either teprotumumab or placebo in a 1:1 ratio. Participants were uniformly within 9 months of the onset of TAO, included both cigarette smokers and non-smokers, and were clinically euthyroid. Once randomized, patients were included in the intention-to-treat (ITT) cohort. Participants in both treatment groups received dosing every 3 weeks for a total of eight infusions over a 24-week intervention phase. The primary response was defined as improvement in the study (worse) eye of both, improvement in CAS of ≥2 points, AND reduction in proptosis of ≥2 mm. This improvement must have occurred in the absence of a similar degree of worsening in the fellow eye. Secondary endpoints, measured as independent variables, included improvement from baseline of CAS of ≥2 points, reduction in proptosis of ≥2 mm, improvement in subjective diplopia, and improved results from a fully validated quality of life questionnaire (GO-QoL). The occurrence and severity of adverse events were also assessed. Any subjects failing to complete the 24-week treatment phase or the assessment at week 24 for any reason were considered treatment failures.

The responses used to judge drug efficacy included reduction in CAS, proptosis, improvement in diplopia, and GO-QoL. These parameters were comparable at the baseline assessment in the two treatment arms. Despite attempts to stratify participants with regard to cigarette smoking, an imbalance in group assignment was detected, with a great number who used tobacco randomized to the placebo group. Analysis of the results demonstrated that of those in the ITT group receiving teprotumumab, 29 of 42 (69%) exhibited a response at 24 weeks. In contrast, nine of 45 (20%) patients receiving placebo responded (p < 0.001). Similar numbers of participants in the two treatment arms completed the intervention phase (87% in the placebo and 88% in the teprotumumab cohorts). The time to response was considerably shorter in the teprotumumab cohort than in those receiving placebo. The onset of response was very rapid. Nearly one-half of those receiving teprotumumab achieved the primary response at week 6, *i.e.* those who had received the half-dose and a single full-dose. The proportion of patients achieving a response was greater in the teprotumumab group and continued to increase throughout the duration of the treatment phase with striking differences compared to placebo which continued to be significant at every time point (all p < 0.001). When the responses were graded, considerably more patients exhibited a high response at week 24 (≥3 point improvement in CAS and ≥3 mm proptosis reduction in the study eye). With regard to the secondary outcomes, those patients receiving teprotumumab responded with a change in proptosis from baseline and CAS from baseline when compared to those receiving placebo. Both responses were significantly different when compared to controls at the 6-week time point (p < 0.001 for both), and the differences continued to increase throughout the treatment phase which ended at week 24 (both p < 0.001). On the basis of this trial, the FDA designated teprotumumab a “breakthrough” therapy for TAO.

### Phase 3 Trial

**Figure 2:**
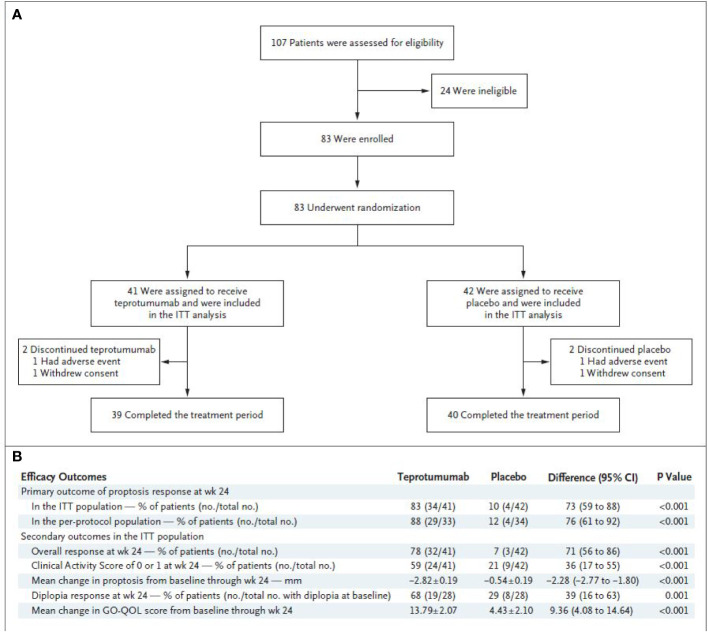
**(A)** Trial profile. **(B)** Efficacy endpoints. * CMH weighting was used to estimate the common risk difference and the 95% Confidence Interval (95% CI) of the common risk difference for the primary and secondary endpoints of overall responder, percent with CAS 0 or 1, and diplopia responder; least squares mean difference was calculated for secondary endpoints of change in proptosis from baseline and change in Graves’ Orbitopathy quality of life (GO-QOL) questionnaire from baseline using the Mixed-Model Repeated-Measures (MMRM) analysis of covariance (ANCOVA) model described below. **^¥^** 28 patients in each treatment group had diplopia at baseline ^‡^ GO-QOL score was calculated and transformed to a 0 to 100 scale. Transformed score = [(sum of each score − number of completed items)/(2 * number of completed items)] * 100 ^§^ Change from baseline in proptosis or GO-QOL as a continuous variable is based on MMRM ANCOVA model with an unstructured covariance matrix including the following terms: baseline score, tobacco use status (non-user, user), treatment group, visit, and visit-by-treatment and visit-by-baseline-score interactions; data presented as least squares mean ± standard error. All analyses were controlled for multiplicity. From N. Engl. J. Med, Douglas R.S, Kahaly G.J., Patel A., Sile E.H.Z., Thompson R et al. Teprotumumab for the treatment of active thyroid eye disease. 382; 341-352. Copyright ^©^ (2020) Massachusetts Medical Society. Reprinted with permission.

A pivotal phase 3 study was initiated shortly after the results of the phase 2 trial had been reported (73). The study was organized and funded by Horizon Pharmaceutical (now Horizon Therapeutics) and included many of the same investigators and institutions participating in the preceding phase 2 trial. It was conducted between October 24, 2017 and August 31, 2018, when 107 patients were screened and 83 underwent randomization (**Figure 2A**). The trial followed an experimental protocol that was very similar to that of the earlier study. It allowed a widened patient age limit (18 years to 80 years) and the inclusion of a study extension, which enabled treatment cross-over for patients failing to respond during the 24-week treatment period and those who had responded initially and then relapsed. Individuals with a history of inflammatory bowel disease were excluded since at least one participating patient in the earlier trial with that diagnosis experienced worsening bowel symptoms. The remainder of the inclusion and exclusion criteria were unchanged from the phase 2 trial. The primary endpoint was proptosis responder rate (percentage of patients with ≥2 mm reduction in the study eye without ≥2 mm increase in the fellow eye) at week 24 for teprotumumab *versus* placebo. Secondary outcomes included overall responder rate (the primary outcome of the phase 2 study) which was defined as the percentage of patients with both ≥2-point reduction in CAS AND ≥2 mm reduction in proptosis in the study eye in the absence of a corresponding worsening in the fellow eye, percentage of patients with a CAS 0 or 1, and diplopia improvement at week 24 and change in GO-QOL overall score through week 24. Unlike the phase 2 trial, this study included an extension. Specifically, those patients who did not achieve the primary outcome *i.e.* the proptosis non-responders, were eligible for participation in an open-label extension study (OPTIC-X (NCT03461211)]. This trial involved eight additional infusions of teprotumumab. Those patients not included in the extension were followed for 48 weeks. Those patients who responded during the initial treatment phase, regardless of treatment arm but then relapsed during follow-up could also enter the extension study.

**Figure 3:**
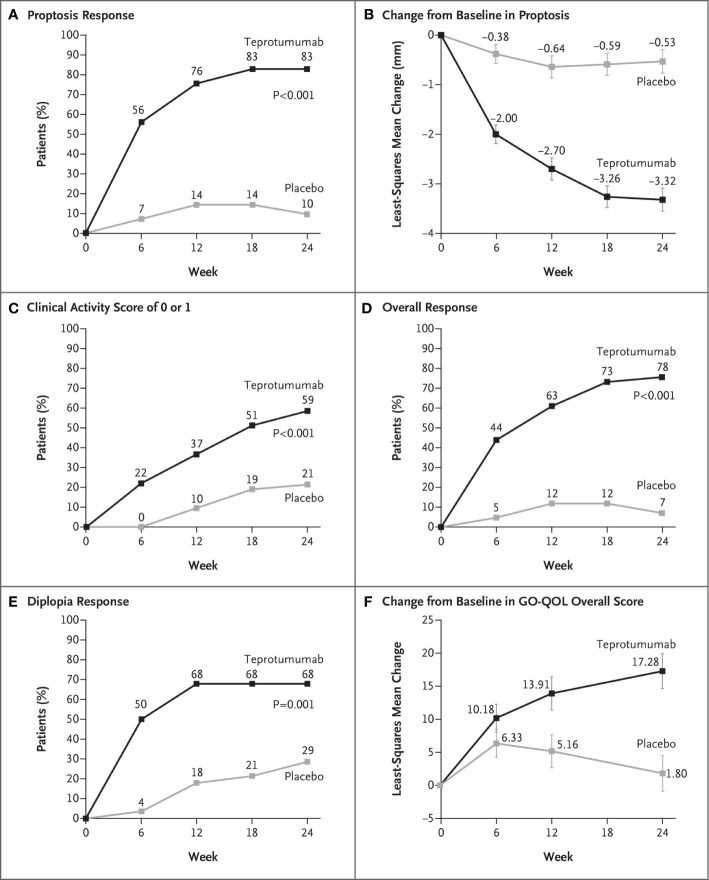
**(A)** Proptosis responder analysis (percent of patients with ≥2 mm reduction in proptosis from baseline in the study eye). **(B)** Change from baseline in proptosis (least squares mean ± standard error). **(C)** Percent of patients with clinical activity score (CAS) of 0 or 1 in the study eye. **(D)** Overall responder rate (percent of patients with ≥2-point reduction in CAS and ≥2 mm reduction in proptosis from baseline in the study eye). **(E)** Diplopia response (percent of patients with improvement of at least one grade from baseline). **(F)** Change from baseline in transformed GO-QOL score (least squares mean ± standard error). From N. Engl. J. Med, Douglas R.S, Kahaly G.J., Patel A., Sile E.H.Z., Thompson R et al. Teprotumumab for the treatment of active thyroid eye disease. 382; 341-352. Copyright ^©^ (2020) Massachusetts Medical Society. Reprinted with permission.

Efficacy outcomes are shown in **Figure 2B**. The results from the trial revealed that 9.5% of patients receiving placebo achieved the primary response compared with 82.9% of those treated with teprotumumab at week 24 (delta 73.45%; 95% CI 58.89 to 88.01%; p < 0.001) (**Figure 3A**). A majority of patients responding did so at week 6. Mean proptosis response from baseline with teprotumumab at week 24 was −3.32 mm *versus* −0.53 in placebo or a mean difference from the placebo group of 2.79 mm (**Figure 3B**).

Secondary outcomes were achieved by significantly more patients receiving teprotumumab than those in the placebo cohort. CAS scores of 0 or 1 (**Figure 3C**) and over-all responders were also more numerous in the teprotumumab group at all study visits (**Figure 3D**). At baseline, both treatment groups included 28 patients with diplopia; improvement of ≥1 diplopia grade was experienced in 50.0% of patients receiving teprotumumab compared to 3.6% of those administered placebo at week 6. Diplopia improved with teprotumumab, regardless of disease severity at baseline when compared to placebo (**Figure 3E**) as did overall quality of life using the GO-QOL score (**Figure 3F**).

**Figure 4:**
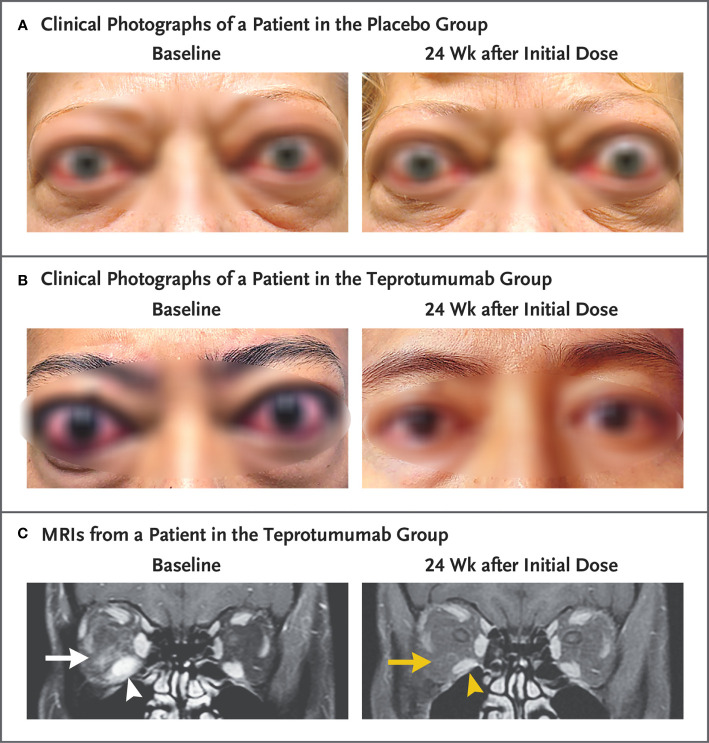
Facial photographic images and MRIs at Baseline and 24 Weeks following treatment with either placebo or teprotumumab. **(A)** Clinical photographs of a patient receiving placebo. At baseline, the patient exhibits substantial proptosis (left eye, 29 mm and right eye, 27 mm) as well as multiple inflammatory signs (left eye Clinical Activity Score of 7 and right eye 5). At week 24, considerable proptosis and inflammatory signs remain. **(B)** Images of a teprotumumab-treated patient. Baseline proptosis (both eyes 24 mm), edema, upper and lower eyelid retraction, and multiple inflammatory signs (CAS 5 bilaterally). At week 24, considerable bilateral reductions in proptosis (−5 mm) and CAS (−4 points). **(C)** Coronal, contrast-enhanced, fat-saturated, T1-weighted MRIs in a single patient receiving teprotumumab at baseline and at week 24. Note marked enhancement of the inferior rectus muscle (white arrowhead) and orbital fat (white arrow) as well as inferior rectus muscle enlargement. At week 24, resolved inferior rectus muscle (yellow arrowhead) enhancement and orbital fat (yellow arrow). The muscle volume was reduced by 49% (yellow arrowhead). Proptosis reduction decreased from 23 mm at baseline to 18 mm at week 24. From N. Engl. J. Med, Douglas R.S, Kahaly G.J., Patel A., Sile E.H.Z., Thompson R. et al. Teprotumumab for the treatment of active thyroid eye disease. 382; 341–352. Copyright ^©^ (2020) Massachusetts Medical Society. Reprinted with permission.

Additional assessments in the phase 3 trial were “before and after” facial photographs (**Figures 4A** and **4B**) and limited orbital imaging performed prior to and following treatment of six patients enrolled (off study protocol) at a single study site. Those imaging studies revealed decreased extraocular muscle volume with reduction of the inferior rectus muscle exhibiting the greatest change in 4/6 patients (**Figure 4C**). Orbital fat volume was reduced in two of these patients. Thus it would appear that teprotumumab affects both extraocular muscle and orbital fat compartments, in at least some patients with TAO.

## Conclusions and Consideration of What Might Lay Ahead for Teprotumumab in TAO and Beyond

In aggregate, the two clinical trials demonstrate the substantial potential for IGF-IR inhibition in the treatment of active, moderate to severe TAO. Because both phase 2 and phase 3 trials excluded TAO of a duration longer than 9 months and did not allow enrollment of individuals with sight-threatening disease, neither study could inform the potential impact of teprotumumab in patients with either long-standing disease or compressive optic neuropathy. Thus, additional studies examining these patients will broaden our insights into the temporal dimensions of benefit and durability the drug can provide. Since its approval by the US FDA in January 2020 (9), case reports have revealed potential effectiveness in clinical stable TAO (74) and in worsening compressive optic neuropathy (75, 76). In addition, the impact of teprotumumab on thyroid function in patients retaining intact thyroid glands should be assessed prospectively. Since other autoimmune diseases, including rheumatoid arthritis, are also associated with abnormalities in the IGF-I/IGF-IR pathway (61, 69), the potential therapeutic effects of IGF-IR inhibition may yield advances in treatments of those conditions as well.

## Author Contributions

The author confirms being the sole contributor of this work and has approved it for publication.

## Funding

This work was supported in part by NIH grants EY08976, Autoimmune Center of Excellence AR088974, and NEI Core grant EY007003.

## Conflict of Interest

TS has been issued patents covering his inventions concerning the use of IGF-IR inhibitors as therapy in Graves’ disease. These patents are held by UCLA School of Medicine and Los Angeles Biomedical Research Institute.
